# Midnolin Ubiquitination is Required for its Proteasome‐Mediated Degradation

**DOI:** 10.1002/mco2.70189

**Published:** 2025-04-24

**Authors:** Jiang He, Tangmin Lai, Yuzu Zhao, Haonan Yang, Zheng Lei, Liu Zhou, Nan Li, Yu He, Wei Zhou, YongZhong Wu

**Affiliations:** ^1^ Radiation Oncology Center Chongqing University Cancer Hospital Chongqing China; ^2^ Department of Breast Cancer Center Chongqing University Cancer Hospital Chongqing China

1

Dear Editor,

The ubiquitin–proteasome system (UPS) is responsible for protein degradation in cells, and proteins to be degraded usually need to be tagged with ubiquitin. Although the proteasome can also degrade proteins that are not tagged with ubiquitin [1, 2, 3, 4, 5], its mechanism has not been fully elucidated. Some important proteins, such as p53, can be degraded through ubiquitin‐independent pathways [5], suggesting that ubiquitin‐independent protein degradation may play a vital role in various biological processes. Midnolin (MIDN) has recently been reported to regulate the ubiquitin‐independent proteasomal degradation of various immediate‐early genes [1], which play crucial roles in wound healing, immune cell activation, as well as neuronal adaptive responses, MIDN can capture proteins through its Catch domain and directly deliver substrates to the proteasome for degradation without substrate ubiquitination. However, whether MIDN can undergo ubiquitination and whether its ubiquitination affects its function remain unclear. Here, our results suggest that MIDN ubiquitination may be required for its proteasome‐mediated degradation.

To explore the function of MIDN, we expressed exogenous Flag tagged MIDN in HEK‐293T cells. We detected the ubiquitination of MIDN after immunoprecipitation using Flag beads, and the results indicated that MIDN is ubiquitinated (Figure 1A), suggesting that MIDN is also regulated by the UPS. Additionally, the ubiquitination level of MIDN significantly increased after treatment with MG132 (Figure 1A), indicating that MIDN ubiquitination may promote its proteasomal functions. However, it is not clear whether the ubiquitination of MIDN affects its proteasome‐mediated degradation.

**FIGURE 1:**
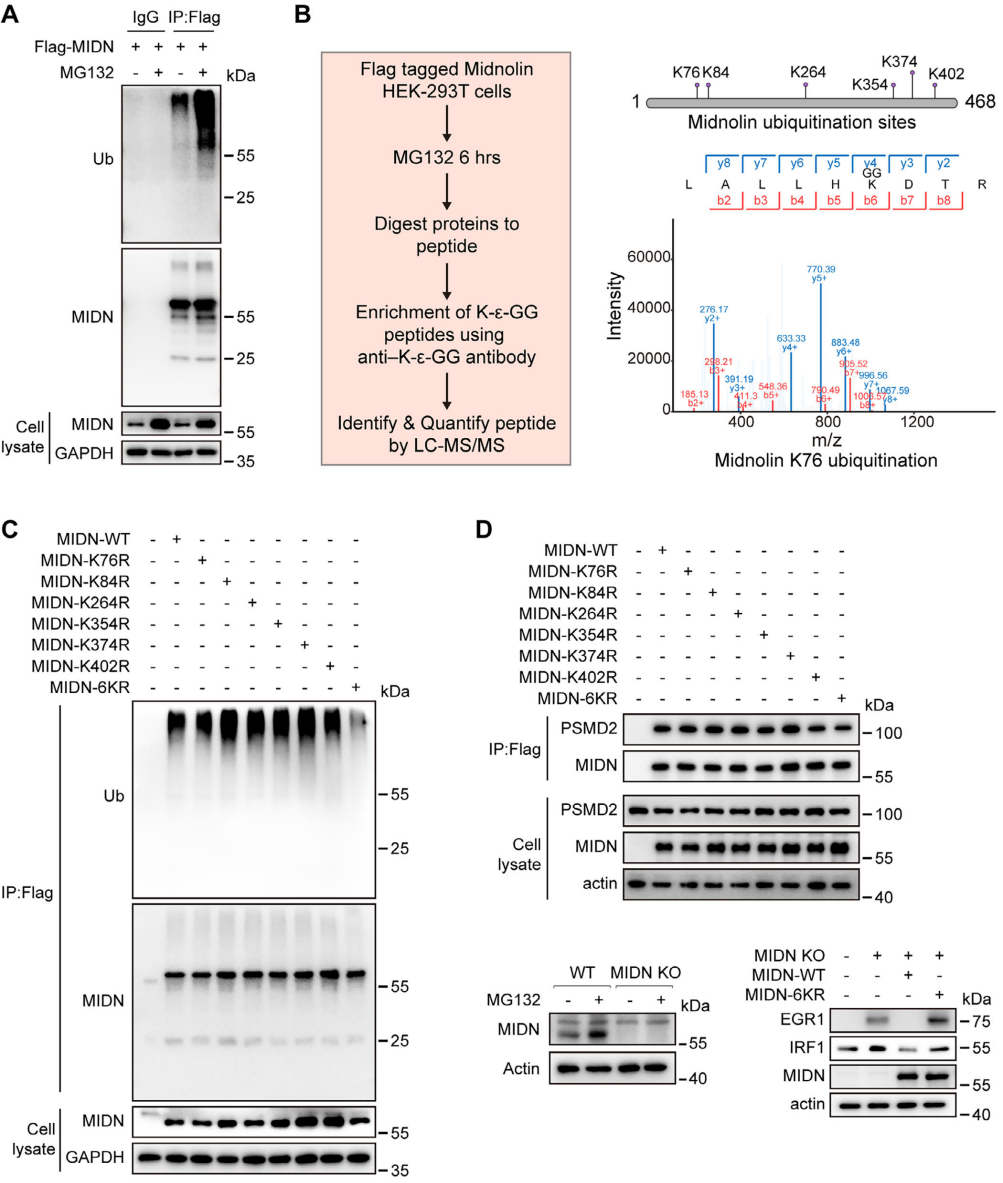
Midnolin is ubiquitinated, and its ubiquitination is required for substrate degradation. (A) HEK‐293T cells expressing Flag‐MIDN were treated with or without MG132 for 6 h before harvesting. Cell lysates were incubated with anti‐Flag Magnetic Beads, IgG Beads were used as a negative control, and the immunocomplexes were immunoblotted with antibodies against ubiquitin (Ub) and Midnolin (MIDN). (B) Diagram for the identification of ubiquitination sites on Midnolin. In brief, the HEK‐293T cells overexpressing Flag‐MIDN were treated with MG132 for 6 h, protein lysates were then prepared to enrich ubiquitinated peptides, and finally, ubiquitination sites were identified through mass spectrometry (left). Illustration of ubiquitination sites on Midnolin. Six potential lysine ubiquitination sites (K76, K84, K264, K354, K374, and K402) in Midnolin identified by mass spectrometry analysis, and a representative mass spectrometry result of Midnolin K76 ubiquitination was shown (right). (C) HEK‐293T cells 293 expressing FLAG‐tagged wild‐type (WT) Midnolin or the K76R, K84R, K264R, K354R, K374R, K402R, and 6KR mutant were pretreated with MG132 for 6 h before harvesting. Cell lysates were incubated with anti‐Flag Magnetic Beads, and the immunocomplexes were immunoblotted with antibodies against ubiquitin (Ub) and Midnolin (MIDN). (D) Coimmunoprecipitation assays with lysates from HEK‐293T cells overexpressing the indicated FLAG‐tagged WT or mutant proteins using anti‐FLAG Magnetic Beads followed by immunoblotting with antibodies against the indicated proteins (upper). Immunoblotting was performed from wild‐type, Midnolin knockout, and Midnolin knockout HEK‐293T cells reconstituted with either WT or 6KR Midnolin. EGR1 and IRF1 are two targets of Midnolin previously reported (lower).

We then identified the ubiquitination sites of MIDN though global proteomic screening of lysine ubiquitination. First, we overexpressed MIDN in HEK‐293T cells and subsequently treated them with MG132 for 6 h. Subsequently, the proteins were digested into peptides, and K‐ε‐GG peptides were enriched using anti‐K‐ε‐GG antibodies (Figure 1B). Finally, liquid chromatography–mass spectrometry analysis revealed that a total of six ubiquitination sites on MIDN, K76, K84, K264, K354, K372, and K402, were ubiquitinated (Figure 1B).

To further investigate the function of MIDN ubiquitination, we constructed Flag tagged mutant proteins with mutations at K76, K84, K264, K354, K372, and K402 with arginine (R), as well as a mutant 6KR with simultaneous mutations at all six sites. We detected the ubiquitination level of each mutant in HEK‐293T cells. The results suggested that the mutation of a single site did not significantly change the ubiquitination level of MIDN (Figure 1C). However, when six sites were mutated simultaneously, the ubiquitination level of MIDN was significantly reduced (Figure 1C). These results suggest that MIDN ubiquitination is not dominated by a single site, but may be coordinated by multiple sites.

Previous studies have established that MIDN promotes its substrates degradation by binding to the proteasome. Therefore, we assessed the binding capacity of these ubiquitination site mutants of MIDN to the proteasome via coimmunoprecipitation assays. The results indicated that whether a single point mutation or a simultaneous mutation of six sites were introduced, the binding of MIDN to the proteasome was not significantly inhibited (Figure 1D). This result implies that abolishing the ubiquitination of MIDN does not affect its ability to bind to the proteasome.

Therefore, we speculated that the ubiquitination of MIDN could potentially influence its capacity for substrate degradation. To substantiate this hypothesis, we generated MIDN knockout cell lines using the CRISPR/Cas9 lentiviral system. Based on previous studies, the protein levels of endogenous MIDN is quite low, and the antibody contains too much background to detect endogenous MIDN protein from cell lysates at steady state. However, pretreating cells with MG132 enables successful detection of endogenous MIDN [1]. Western blot analysis revealed successful depletion of MIDN (Figure 1D). According to previous reports, EGR1 and IRF1 are two substrates of MIDN [1]. We detected the expression levels of these two proteins in the MIDN knockout cells, and the results showed that after MIDN knockout, the expression of its substrates EGR1 and IRF1 was upregulated, while overexpression of wild‐type MIDN could increase its degradation of these substrates, while overexpression of the 6KR mutant inhibited its degradation of substrates (Figure 1D). These results suggest that the degradation of MIDN on its substrates may depend on its ubiquitination (Supporting Information).

Overall, our investigation reveals that MIDN exhibits ubiquitination. Then, we identified the ubiquitination sites of MIDN by global proteomic screening of lysine ubiquitination. We found that MIDN 6KR mutant has impaired function in its degradative ability but do not affect its binding to the proteasome. The UPS is an important protein degradation pathway, and the regulatory mechanism of MIDN provides a new perspective on the UPS and ubiquitin‐independent protein degradation. Since many proteins may undergo ubiquitin‐independent degradation, although this process does not require the ubiquitination of the substrate, the ubiquitination of these regulatory factors may play a functional role in ubiquitin‐independent degradation. MIDN ubiquitination contributes to its function and adds a new perspective on how the UPS works.

## Author Contributions

J. H., T. L., and Y. Z. cowrote the manuscript. J. H., T. L., Y. Z., H. Y., Z. L. L. Z. N. L., and Y. H. conducted the experiments and/or analyzed the data. W. Z. and Y. W. conceived this project. All authors have read and approved the final manuscript.

## Ethics Statement

No specific approval was needed for this study.

## Conflicts of Interest

The authors declare no conflicts of interest.

## Supporting information



Supporting Information

## Data Availability

The data are available from the corresponding author upon reasonable request.
