# Supplementary material for: Midnolin Ubiquitination is Required for its Proteasome‐Mediated Degradation
Source: MedComm (2020). 2025 Apr 24;6(5):e70189. doi: 10.1002/mco2.70189 (PMC12022415; doi:10.1002/mco2.70189)
Supplement: Supplementary file 1 — Supporting Information [file MCO2-6-e70189-s001.docx]

**SUPPLEMENTAL INFORMATION**

**Midnolin** **ubiquitination is required for its proteasome-mediated degradation**

Jiang He^1,#^, Tangmin Lai^1,#^, Yuzu Zhao^2,#^, Haonan Yang^1^, Zheng Lei^1^, Liu Zhou^1^, Nan Li^1^, Yu He^1^, Wei Zhou^1,^*, YongZhong Wu^1,^*

^1^Radiation Oncology Center, Chongqing University Cancer Hospital, Chongqing 400030, China.

^2^Department of Breast Cancer Center, Chongqing University Cancer Hospital, Chongqing 400030, China.

^#^Jiang He, Tangmin Lai and Yuzu Zhao contributed equally to this work.

*Correspondence

Wei Zhou and YongZhong Wu, Radiation Oncology Center, Chongqing University Cancer Hospital, Chongqing University, No. 181 Hanyu Road, Shapingba District, Chongqing 400030, China.

E-mail: [zhouwei978978@163.com](mailto:zhouwei978978@163.com) (W.Z.); [yongzhong.wu@cqu.edu.cn](mailto:yongzhong.wu@cqu.edu.cn) (Y.W.)

**SUPPLEMENTAL EXPERIMENTAL PROCEDURES**

**Materials and Methods**

**Immunoprecipitation**

For immunoprecipitation analysis, cells were plated in 100-mm plates and then lysed in western blot and immunoprecipitation lysis buffer (Beyotime). Whole-cell lysates were incubated with Anti-Flag Magnetic Beads (Sigma) at 4°C overnight. Following extensive washing in phosphate buffered saline (PBS), bound proteins were recovered by boiling the beads in 1× sodium dodecyl sulfate–polyacrylamide gel electrophoresis sample loading buffer.

**Cell culture and transfection**

HEK-293T cell lines were purchased from ATCC, all cell lines used in this study have undergone authentication by short tandem repeat (STR) analysis to confirm their identity and tested mycoplasma free. Dulbecco’s modified Eagle’s medium (DMEM) contained 10% FBS and 1% P/S was used for maintaining HEK-293T cells. All cell lines were cultured in the 5% CO2 incubator at 37℃ with saturated humidity. Transfection was performed with Lipofectamine 2000 as described in manufacturer’s instruction.

**Lentiviral CRISPR-Cas9 plasmids and cloning**

To construct lentiviral CRISPR-Cas9 vectors with the desired sgRNA, the lentiCRISPRv2 backbone (Addgene, 52961) was initially digested using BsmBI (NEB, R0739S). Subsequently, sgRNA oligos featuring CACC or AAAC overhangs were synthesized by Shenggong, phosphorylated, annealed, and ligated into the linearized backbone using T4 ligase (NEB, M0202S). The sgRNA sequences used were as follows: nontargeting sgControl: GTATTACTGATATTGGTGGG; human sgMIDN: GAAGCTGCAGGAGTTCGGCG. To validate Midnolin knockout efficiency, cells were pretreated with 10 µM MG132 for 6 hours.

**Western Blot**

Cells were collected and then lysed in a RIPA lysis buffer (Beyotime) with Phenylmethanesulfonyl fluoride (Beyotime). Cell lysates were denatured at 100 °C for 10 min. 10% SDS-PAGE gels were used to separate proteins. Proteins were then transferred to polyvinylidene difluoride membranes. After blocking membranes in 5% nonfat dry milk at room temperature for 1 h. Primary antibodies against Ub (1 : 1000, Proteintech Group), MIDN (1 : 1000, Proteintech Group), PSMD2 (1 : 1000, Proteintech Group), Actin (1 : 5000, Cell Signaling Technology), EGR1 (1 : 1000, Cell Signaling Technology), and IRF1 (1 : 1000, Cell Signaling Technology) were incubated with membranes at 4 °C overnight. Membranes were incubated with horseradish peroxidase-conjugated secondary antibody (HRP-conjugated secondary antibodies, goat anti-mouse IgG or goat anti-rabbit IgG, 1 :5000, Cell Signaling Technology) at room temperature for 1 h. Proteins were finally visualized by ECL system (Beyotime) and then captured by ChemiDoc Imaging System (BIO-RAD).

**Data availability**

The raw data of ubiquitination proteomics can be obtained via Pride.
